# Acute hematogenous osteomyelitis of the talus: a case report

**DOI:** 10.11604/pamj.2020.37.232.23502

**Published:** 2020-11-13

**Authors:** Nabil Dammak, Aymen Hannafi, Hassen Cheikhrouhou, Maher Teka, Hazem Ben Ghozlen, Faouzi Abid

**Affiliations:** 1 University of Monastir, Taher Sfar Hospital of Mahdia, Department of Orthopaedic Surgery, Mahdia 5100, Tunisia

**Keywords:** Acute, hematogenous, osteomyelitis, talus, case report

## Abstract

In children, osteomyelitis is common in the long bones of the femur and the tibia. However, talar osteomyelitis is extremely rare. The condition is difficult to diagnose due to the slow onset and atypical pattern of the symptom. We report the case of a 9-month-old male, coming from a rural area, with talar osteomyelitis. The patient's history of a trauma that resembled an ankle contusion had delayed the diagnosis and subsequently developed into septic arthritis. He was treated by curettage, immobilization in plaster and appropriate antibiotics with full recovery of function in his right ankle and foot three months after the first outpatient visit. The bony cavity was healed six months after the operation and there were no growth disturbances or any abnormalities of the adjacent joints. This case report aims to present the difficulties in the diagnosis and therapeutic approach of this disease.

## Introduction

Osteomyelitis is a common occurrence in the pediatric population and typically involves the metaphysis of long bones but can involve atypical locations such as the talus (less than 1%). In localizations like the talus, the diagnosis of osteomyelitis is usually challenging and made late, mostly because of the less pronounced symptoms compared with in long-bone localizations [1,2]. This case report aims to present the difficulties in the diagnosis and therapeutic approach of this disease.

## Patient and observation

**Figure 1:**
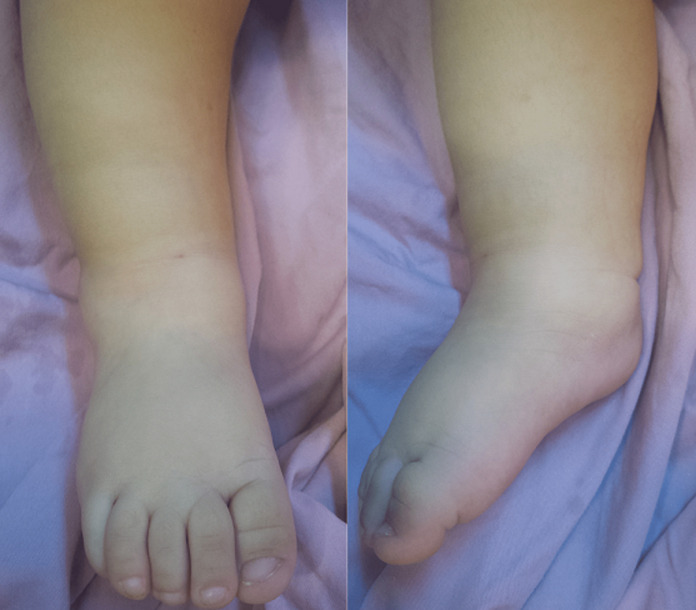
clinical aspect observed at admission

**Patient information:** a 9-month-old boy presented to the emergency room with a 3-day history of refusal to bear weight on the right lower extremity and febrile peaks of up to 38.5°C for 24 hours. His parents had noted an ankle trauma in the previous week. The primary care physician had initially suspected a talus fracture. His right ankle was immobilized by a plaster splint and his parents were instructed to apply ice. However, the child continued to experience ankle pain and fever. He was returned to the emergency room after two days. His symptoms were aggravated. The ankle was noted to be slightly edematous, but warm to the touch, and diffusely tender to palpation (Figure 1). His temperature was 39°C.

**Figure 2:**
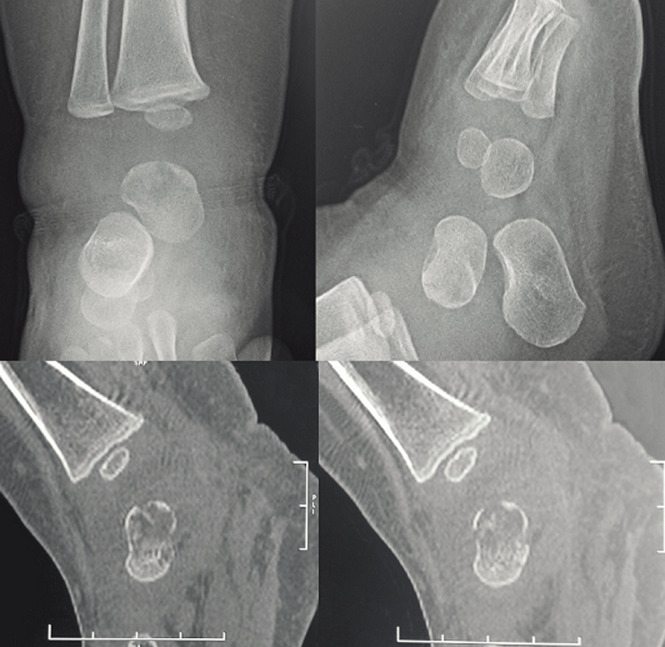
ankle imaging: X-ray of the ankle showing a lytic lesion of the talus; CT scan showing a talus fracture

**Diagnostic assessment:** the hemoglobin was 9.5 g/dl, the white blood cell count was 18800/mm^3^, and the C-reactive protein (CRP) was 75 mm/h. Serum laboratory studies, including CBC count and inflammatory markers, were all elevated. An X-ray of the ankle showed a posterior-medial lytic lesion of the talus and soft tissue swelling around the ankle joint. However, the computed tomography (CT) scan findings revealed a talus fracture (Figure 2). A magnetic resonance imaging (MRI) was not done as it was not available. Given the clinical findings a talar osteomyelitis with septic arthritis of the ankle was suspected despite the result of the CT scan. The bone scintigraphy demonstrated a septic arthritis and the presence of bone marrow edema in the talus without being able to reach a precise diagnosis of osteomyelitis.

**Figure 3:**
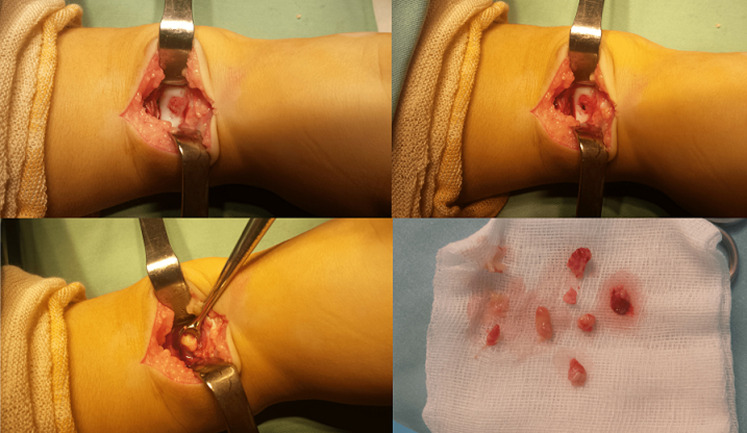
per-operative exploration: pertuit at the junction bone cartilage of the talus; drainage of organized fibrinous pus

**Therapeutic intervention:** the patient was urgently taken to the operating room. During the operation, the ankle joint was explored through an anterior approach. There were some false membranes, a thin turbid fluid and a pertuit at the junction bone cartilage of the talus. The talar cartilage was regular and normal-looking (Figure 3). The pertuit, allowing access to a cavity, was cleaned and the yellow organized fibrinous pus was drained (Figure 3). The biopsy specimens for the histopathological examination and culture were taken at that site. Curettage and irrigation were performed, and the joint was closed over suction drains. An empirical antibiotic treatment IV (cephalosporin + gentamycine) was initiated.

**Figure 4:**
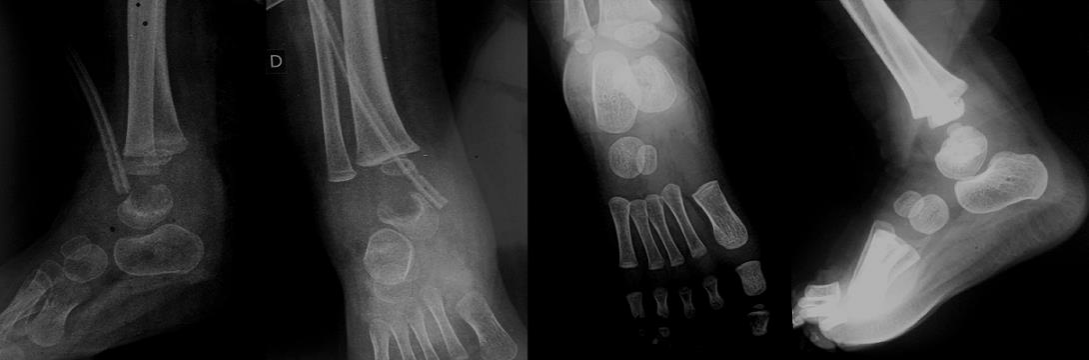
filling of the lesion 6 months after surgery

**Follow-up and outcomes:** febrile peaks persisted during the first three days. The blood culture and the specimen of the joint isolated *Staphylococcus aureus* Meti-R. The anatomopathological analysis of the ankle specimen showed histological features of an abscess and hyperplastic synovial cells. So, the IV antibiotic treatment was substituted by teicoplanin with a better clinical and biological progression. Drainage was maintained for twelve days (until normalization of CRP) and intravenous antibiotics for 3 weeks. In the third week, the peripheral leukocytes count was 8600/mL, and the CRP decreased to 2.43mg. The patient's antibiotic was then substituted by oral pristinamycin for three weeks. In the fourth week, the ESR and CRP were 7 mm/hr and 3 mg/L, respectively. At that time, the patient did not complain of any pain or limitation of the range of motion of the ankle. The cast was removed six weeks after surgery. At six months, radiographies showed that the lesion healed completely (Figure 4). The parents were very satisfied. Their child acquired walking at the age of 14 months. In the last follow-up visit at 18 months postoperatively, the patient had no pain or limitation in walking and running.

## Discussion

Osteomyelitis (OM) is an inflammation of the bone. The condition is usually due to infection with bacteria or other micro-organisms and is associated with bone destruction. It is commonly divided into three types based on etiology: hematogenous, secondary to a contiguous infection, and secondary to direct inoculation. The analysis of its skeletal distribution showed that the metaphyses are the sites affected most commonly due to the characteristic circulation and the presence of sinusoids, mainly in the femur and tibia [3]. If the infection in the metaphysis is not been efficiently controlled or treated, it can spread into the epiphysis and produce concurrent septic arthritis because the capsule of the joint often extends to the metaphysis until 15 to 18 months of age [4]. Tubular bones areoften involved. However, isolated osteomyelitis of the talus in a child is a very rare incidence. It makes up less than 1% of all cases of pediatric osteomyelitis. This diagnosis can be easily missed because of its rarity and the absence of evident clinical manifestations as in older children and adults. A delayed diagnosis can result in complications such as bone deformity and chronic osteomyelitis [5].

Imaging always starts with conventional radiographs because of their low cost, availability, and ability to suggest correct diagnosis and exclude other diagnoses [6]. The first radiographic sign of Acute Hematogenous Osteomyelitis (AHO) is deep soft tissue swelling with lytic changes in the bone. The MRI remains the gold standard for diagnosis of acute OM with a sensitivity and specificity of 91% and 82% respectively. It has been shown to detect OM as early as up to five days from the start of the infection. The MRI is also useful in the evaluation of complications such as abscesses, joint effusions, and soft tissue extensions that may require surgery [7]. Multiple acute-phase reactants have been described and evaluated for their utility diagnosing infection, including C-reactive protein (CRP), erythrocyte sedimentation rate (ESR), white blood cell count (WBC), interleukin 6, and D-dimer [8]. CRP and ESR have proved the most useful in evaluating AHO. In children younger than 4 years old, 60% of cases of infection are related to Streptococcus and Gram-negative bacteria [4]; the remaining case is due to *Staphylococcus aureus*.

In their research study, Howard-Jones *et al*. concluded: “We suggest that uncomplicated acute osteomyelitis in children >3 months old should be treated with 3-4 days of IV antibiotics, and if the child is responding clinically, they can transition on oral antibiotics to a total duration of 3 weeks”. The antibiotic choice in many of the studies was initially empirical with intravenous (IV) antibiotics to cover methicillin-sensitive *Staphylococcus aureus* and then later switched to a pathogen-specific antibiotic based on culture results. A temperature > 38.4°C and a CRP value > 100 mg/L are the best indicators of the need for continuing the IV treatment [9]. Surgical management with early biopsy and debridement is now used less frequently than in the past, and there is a tendency to employ conservative treatment. However, surgery is vital in case of complicated OM or associated arthritis [10]. In front of a discrepancy between the clinico-biological characteristics of osteoarticular infection and inconclusive radiological explorations, we must indicate surgery for the exploration and collection of bacteriological and anatomopathological samples.

## Conclusion

Delay in the diagnosis and treatment of osteomyelitis affecting the talus may put the child at risk of developing chronic and debilitating complications. Emphasis should be placed on earlier diagnosis to improve prognosis and prevent sequelae.
